# Inferior Vena Cava Filter Fracture: Potential Liability for Emergency Physicians

**DOI:** 10.5811/westjem.2015.1.25326

**Published:** 2015-03-06

**Authors:** Richard M. Pescatore, Brigitte M. Baumann, David Nocchi

**Affiliations:** Cooper University Hospital, Department of Emergency Medicine, Camden, New Jersey

## INTRODUCTION

**Figure 1:**
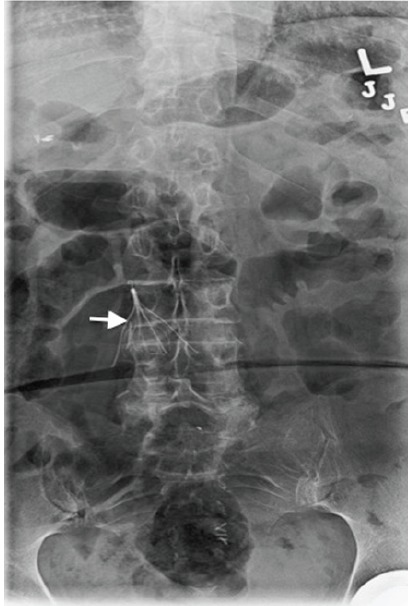
Posterior to anterior view of lumbosacral spine. Arrowhead demonstrates fracture and displacement of the posterolateral inferior vena cava filter leg.

**Figure 2:**
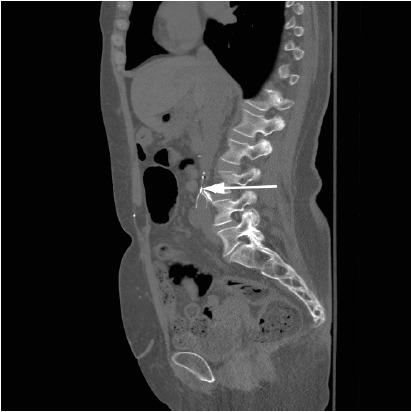
Sagittal computed tomography view of lumbosacral spine. Arrowhead demonstrates fracture and displacement of the posterolateral inferior vena cava filter leg.

A 58-year-old female presented to the emergency department complaining of low back pain following a motor vehicle crash. She denied loss of consciousness, headache, or extremity weakness. Her past medical history was notable for inferior vena cava (IVC) filter placement many years ago due to multiple pregnancy-associated deep venous thromboses (DVTs). Physical exam was unrevealing: no spinal point tenderness and a nonfocal neurologic exam. Posteroanterior lumbosacral radiograph demonstrated fracture and displacement of the posterolateral IVC filter leg (Figure 1). Computed tomography showed the filter leg to be perforated through the IVC (Figure 2). After consultation with the interventional radiologist, it was decided that the risks of retrieval outweighed potential benefits, and the patient was discharged for outpatient monitoring of the fractured limb.

## DISCUSSION

From 2000–2009, the number of IVC filters placed in the United States increased from 56,380 to 132,049, with the majority in patients with a pulmonary embolism or DVT. Paralleling this increase, a rise in complications is also anticipated, including IVC filter migration, embolization and strut fracture.^1^ The incidence of IVC filter strut fracture ranges from 2% to a predicted 40% at 5.5 years.^2^,^3^ Strut fracture may predispose an IVC filter or a portion thereof to embolize and may also alter flow mechanics, decreasing the IVC filter’s ability to prevent a pulmonary embolism.^1^ In 2010, the U.S. Food and Drug Administration issued warnings regarding the safety of IVC filters after life-threatening complications occurred from filter fracture and embolization of filter limbs, including ventricular wall laceration resulting in cardiac tamponade and tachycardia induced by cardiac irritation.^4^,^5^ Given these risks, failure to identify and refer patients with IVC filter fracture for further evaluation and potential retrieval may represent a potential liability for emergency physicians in the event of embolization and subsequent injury.^6^
